# OA06.01. A randomized controlled trial of 8-form Tai chi improves symptoms and functional mobility in fibromyalgia patients

**DOI:** 10.1186/1472-6882-12-S1-O21

**Published:** 2012-06-12

**Authors:** S Mist, K Jones, C Sherman, J Carson, R Bennett, F Li

**Affiliations:** 1 Oregon Health & Science University, Portland, USA; 2 Oregon Research Institute, Eugene, USA

## Purpose

Previous researchers have found that 10-form Tai Chi yields symptomatic benefit in patients with fibromyalgia (FM). The purpose of this study was to further investigate these findings and focus on functional mobility.

## Methods

We conducted a parallel-group randomized controlled trial for FM using a modified 8-form Yang-style Tai chi program compared to an education control. Participants met in small groups twice weekly for 90 minutes over 12 weeks. The primary endpoint was symptom reduction and improvement in self-report physical function, as measured by the Fibromyalgia Impact Questionnaire (FIQ), from baseline to 12 weeks. Secondary endpoints included pain severity and interference [Brief Pain Inventory (BPI)], sleep (Pittsburg Sleep Inventory), self-efficacy and functional mobility.

## Results

Of the 98 randomly assigned subjects (mean age 54 years, 93% female), those in the Tai chi condition compared to the education condition demonstrated clinically and statistically significant improvements in FIQ scores (16.5 vs. 3.1, p<0.0002), BPI pain severity (1.2 vs. 0.4, p<0.0008), BPI pain interference (2.1 vs. 0.6, p<0.0001), sleep (-2.0 vs. -0.03, p<0.0003) and self-efficacy for pain control (9.2 vs. -1.5, p<0.0001). Functional mobility variables including timed get-up and go (-0.92 vs. -0.25, p<0.0001), static balance (7.5 vs. -0.3, p<0.0001) and dynamic balance (1.6 vs. -0.5, p<0.0001) were significantly improved with Tai chi compared to education control. No adverse events were reported.

## Conclusion

Tai chi appears to be a safe and effective mind/body exercise treatment that could be used as an adjunctive modality in FM patients for both symptom reduction and functional mobility improvement. (ClinicalTrials.gov Identifier, NCT01311427).

